# Influence of the Nanotube Morphology and Intercalated Species on the Sorption Properties of Hybrid Layered Vanadium Oxides: Application for Cesium Removal from Aqueous Streams

**DOI:** 10.3390/nano11092349

**Published:** 2021-09-10

**Authors:** Delhia Alby, Fabrice Salles, Jerzy Zajac, Clarence Charnay

**Affiliations:** Institut Charles Gerhardt (ICGM), Univ Montpellier, CNRS, ENSCM, 34090 Montpellier, France; delhia.alby@umontpellier.fr (D.A.); fabrice.salles@umontpellier.fr (F.S.)

**Keywords:** sorption, cesium, aqueous solutions, layered vanadium oxides, nanotube morphology, interlayer species, hexadecylammonium and ammonium cations

## Abstract

The present paper examines the impact that the nanotube morphology and organic or inorganic intercalated species may have on the cesium sorption by layered vanadium oxides prepared with the use of hexadecylamine as a structure-directing agent. The hybrid material represented by a chemical formula of (V_2_O_5_)(VO_2_)_1.03_(C_16_H_36_N)_1.46_(H_2_O)_x_ was achieved through accelerated microwave-assisted synthesis carefully optimized to ensure the best compromise between the scroll-like morphology and the hydrophobic character. To enhance its dispersibility in water, this sample was subsequently modified by progressive replacement of the C_16_H_36_N^+^ units by NH_4_^+^ cations. The final materials represented a stacking of lamellar sheets with a worse scroll-like morphology. Both the optimization procedure and the template removal were monitored on the basis of scanning and transmission electronic microscopy, X-ray diffraction, infra-red spectroscopy, inductively coupled plasma-optical emission spectrometry, X-ray photoelectron spectroscopy, and elemental analysis, supplemented by adequate simulations methods providing the reference IR spectra and XRD patterns for comparison or the textural parameters of the samples. The comparison of the cesium sorption from either a 4:1 ethanol–water mixture or aqueous solutions pointed toward the solubility of intercalated cations in the bulk solution as the main factor limiting their displacement from the interlayer space by the oncoming cesium ones. The sample obtained after 70% exchange with NH_4_^+^ exhibited a maximum sorption capacity of 1.4 mmol g^−1^ from CsNO_3_ aqueous solutions and its retention efficiency remained significant from low-concentration Cs solutions in river or sea water.

## 1. Introduction

Sorption processes occurring at the solid–liquid interface have gained substantial importance in remediation of liquid waste streams over the past decades since, in addition to fast operational readiness and easy handling, they may offer low-level discharge generation and significant reversibility of the removal process [1,2,3]. Among various types of pollutants present in liquid streams, heavy metals are usually ranked as the top listing on the Priority List of Hazardous Substances of the Agency for Toxic Substances and Disease Registry. In the particular case of radionuclides, their release to the environment, even at very low quantities, greatly increases environmental and health risks. The in situ removal of heavy metals from industrial wastewater still remains the first priority for the international scientific and technical community [4,5,6,7,8,9,10,11], though the sorption technology has attracted increasing attention also in the field of ethanol purification for fuel usage [12,13] or even in the elimination of heavy metals from ethanol extracts [14]. In many such cases, organic-mineral oxide hybrid materials are proposed, which should combine good removal efficiency and selectivity with sufficient dispersibility in appropriate liquid media. In consequence, the research and preparation of materials in relation to these challenges are guided by the necessity of adapting the sorption properties of the designed adsorbents to the particular retention mechanism for each individual metal pollutant under given operating conditions.

When a given metal pollutant occurs in the form of free or complex ions in aqueous solutions, its retention on solid surfaces is affected by the concomitant changes in the structure of the ionic double layer formed at the interface as a function of the pH and ionic strength [15,16]. The sorption phenomenon thus has a strongly competitive character and the selectivity of its action depends on the charge and size of competing ionic species, as well as their specific interactions with the surface. Taking account of the size aspect, the idea of exploiting structural flexibility of inorganic lamellar materials to modulate the retention selectivity toward heavy metal cations has appeared very fruitful, especially in the case of ^137^Cs or ^90^Sr radionuclides [17,18,19,20,21]. Indeed, the lamellar structures can swell or shrink, since the cohesive forces between the layers are weak, thus allowing the interlayer space to adjust easily to the dimension of the inserted species. Furthermore, inorganic materials have the advantage over organic resins due to their capability of enduring radiation and high temperature conditions while still keeping a high ion exchange capacity [18,19,22,23,24]. Some may even constitute the basis for the formation of mineral matrices allowing isotope’s immobilization and their storage in landfills [25,26,27].

In light of all these arguments, it appears to be justified that nanostructured inorganic materials have received so much attention with respect to the removal of radioactive strontium or cesium [28]. Here, the importance of the distinct morphology and nanostructure of sorption materials in determining their retention capacity toward such radionuclide cations may be the subject of a debate. One outstanding example is provided by titanate nanomaterials where titanate nanotubes have shown better retention efficiency than other types of nanostructures [19,29,30].

It is within this framework that hybrid nanomaterials achieved on the basis of vanadium oxide nanotubes were tested in the present study. The synthesis of vanadium oxide nanotubes achieved by periodic 2D layer scrolling when making use of different amines as the structure-directing templates was reported in numerous papers [31,32,33,34,35,36,37]. These nanotubes were demonstrated to represent nanorolls of defect-rich VO_x_ sheets with an excess of negative charges compensated by cations intercalated in the interlayer region. The driving force of vanadate scrolling was argued to be related to a partial reduction of V^5+^ to V^4+^ induced by the interaction of amine units with the 2D vanadate layers. In this respect, they may be potentially considered as ion exchangers for the capture of metal cations from wastewater, though the removal of the organic template was postulated to be necessary to render the materials dispersible in aqueous media [36].

In our previous paper [36], layered vanadate oxides with a scroll morphology were prepared by microwave-assisted hydrothermal synthesis while referring to such templating amines as 3-phenyl-1-propylamine, heptylamine, decylamine, dodecylamine, didecylamine, hexadecylamine, and stearylamine. The cesium capture from single-solute solutions in ultrapure water was argued to exhibit a clear relationship with the amine structure through the surface charge density. The latter was found to be significantly increased in the case of amines with moderate chain lengths which could interact more with the layer surface upon synthesis, thereby enhancing the vanadium reduction from +5 to +4. Simultaneously, the exchange of amine units with ammonium cations, necessary to underline the cation retention action of the materials in aqueous streams, was much easier for the same amines. On the other side, the nanotube morphology was more perfect with amine templates containing long chains. Furthermore, the presence of amine units within the interlayer space appeared to affect the structuration of the vanadate layers through decreasing the turbostratism, which resulted in better accessibility of sorption sites within pores.

In light of the above consideration, vanadium oxide nanotubes achieved by using hexadecylamine as the structure directing agent were selected for the purpose of the present study. A considerable optimization work was done to prepare homogenous nanotubes possessing a scroll-like morphology by referring to microwave-assisted hydrothermal synthesis route in which the following parameters were varied: vanadium-to-amine molar ratio, aging time, and duration of the hydrothermal treatment. The cesium retention capacity of one well-structured hybrid material was subsequently tested in single solute solutions in both ethanol–water mixture and ultrapure water. Some indications about the plausible retention mechanism were given on the basis of elemental analysis of the solid sample loaded with cesium. These results also pointed out the necessity of, at least, partial displacement of amine from the interlayer space so as to enhance its sorption performance in aqueous streams. This hybrid material was thus modified through a gradual substitution of the amine template units by ammonium cations. A combination of classical structural characterization and DFT (Density Functional Theory) calculations provided insight into the structural modification of the materials triggered by the removal of the organic template. The retention performance of the best-structured hybrid sample in terms of its sorption capacity and affinity toward cesium was assessed in single solute aqueous solutions and multi-component solutions simulating the mean composition of river and sea water. This variety of liquid media constituted originality of the present study and allowed the action of the materials to be tested in a larger spectrum of cases.

## 2. Materials and Methods

### 2.1. Materials

Vanadium(V) pentoxide (purity 99.2%) was purchased from Alfa Aesar (Thermo Fisher Scientific, Kandel, Germany) and hexadecylamine (>94%) from Merck Millipore (Molsheim, France). Ammonium nitrate (>98%), hexane (>99%), and cesium nitrate (99%) were Sigma-Aldrich (St. Quentin Fallavier, France) products. Absolute anhydrous ethanol (>99.9%) was acquired from Carlo Erba (Val-de-Reuil, France). The 18.2 MΩ cm ultrapure water (PURELAB^®^ Chorus 1, ELGA Veolia, France) was used to prepare cesium solutions. The two types of multicomponent aqueous solutions used as mean solvents to simulate the composition of river and sea waters are reported in Table 1.

### 2.2. Preparation of Vanadium Oxide Nanotubes

Vanadium oxide nanotubes were obtained through a hydrothermal synthesis under microwave radiation in a Teflon autoclave with the StartSynth^®^ oven from Milestone Company (Paris, France). The magnetron delivered a power up to 1200 W (2400 Hz) and the temperature was controlled by an optical fiber system. The synthesis procedure was adapted from a conventional hydrothermal synthesis of vanadate nanotubes in a classical oven [31,37]. In a typical synthesis, vanadium pentoxide and hexadecylamine (molar ratio of 1:0.8) were mixed in ethanol (25 mL) under constant stirring for 2 h. The yellow suspension was then hydrolyzed with ultrapure water (30 mL), thus inducing the formation of a compact gel changing subsequently into a dense solution during maturation. After aging (3 days), the compound thus obtained was transferred into a 100 mL Teflon autoclave for hydrothermal treatment in microwave furnace at 463 K during 2 h. The temperature was increased to a desired value by providing a heating power of 500 W for 10 min. After cooling down to room temperature, the resulting solid was washed with ethanol and hexane and finally dried at 343 K in a classical oven. 

In order to decrease the hydrophobic character of the material imposed by the organic template, the hybrid nanotubes issued directly from the synthesis were submitted to a repeated treatment based on the removal of hexadecylamine by ammonium nitrate [36,39]. Simultaneously, this procedure allowed the cation exchange capacity of the hybrid material to be evaluated in both quantitative and qualitative ways. For that purpose, 3 g of the material was placed in 300 mL of an ethanol–ultrapure water (volume ratio of 4:1) solution under reflux for 5 h at 373 K. The so-obtained compound was then washed successively in ethanol and water. After each washing step, the solid was recovered by centrifugation and then dried at 343 K. The washing-centrifugation-drying procedure was repeated 2 or 3 times in order to decrease the final amount of amine and increase the vanadium-to-amine molar ratio.

### 2.3. Characterization of Vanadium Oxide Nanotubes

The wide-angle X-ray diffraction (XRD) patterns measured at room temperature either in ambient air or in vacuum were obtained by means of a Philips X’pert θ-θ diffractometer using Cu Kα radiation (λ = 1.5418 Å) with a power source of 45 kV and 30 mA. The small-angle diffraction patterns (2θ from 1.5° to 10°) were recorded with a 1/16 slit. The shape and morphology of the synthesized samples were investigated by scanning electron microscopy (SEM) by means of a FEI Quanta 200 FEG microscope with a high-resolution field emission, as well as by transmission electronic microscopy (TEM). The analyses were realized on a JEOL 1200EX2 equipped with a CDD captor of 11 Mpixels (SIS Olympus camera Quemesa model) and an acceleration tension of 100 kV. 

The contents of carbon and nitrogen in a given sample were determined by combustional elemental analysis with the aid of an Elementar vario MICRO cube equipped with MX5 Comparator Microbalance (Viroflay, France). The powdered sample was first wrapped in a small tin foil. The tin foil was then introduced into a combustion tube of the elemental analyzer where it was fully combusted in the presence of oxygen. In a subsequent step, the gases thereby generated were reduced and excess oxygen was removed, thus leaving only such simple gases as CO_2_, H_2_O, and N_2_. These gases were then collected and analyzed for each element and the results were presented as weight percents of the total sample mass. 

X-ray photoelectron spectroscopy (XPS) was used to obtain the metal oxidation state and the surface composition of the selected samples. The photoelectron spectra acquired from ESCALAB 250 spectrophotometer (Thermo Electron, Villebon-sur-Yvette, France) were calibrated with respect to the bond energy in agreement with the energy of the C-C component from the carbon C(1s) at 284.8 eV and a monochromatic excitation source (Al K_α_ 1486.6 eV) with a spot diameter of 400 µm to analyze the sample surface. 

The metal content in weight percent was corroborated by inductively coupled plasma-optical emission spectrometry (ICP-OES) with a Perkin Elmer Optima 7000DV. The apparatus was equipped with a cyclonic spray chamber and a Meinhard nebulizer operating at a flow rate of 0.80 L min^−1^. The radiofrequency power was set at 1300 W. Prior to the ICP-OES measurements, the solid sample was dissolved in concentrated nitric acid and diluted in water. The wavelength used for the analysis of vanadium was equal to 310.273 nm (3 replicates).

Infrared spectroscopy study was done in reflectance mode on a Frontier MIR Spectrometer with a Pike GladiATR. Infrared (IR) spectra were recorded between 4000 and 500 cm^−1^ on samples dispersed in potassium bromide (KBr) pellets (4 wt.%). 

### 2.4. Sorption Performance Tests in Polar Liquid Solutions

A test of cesium retention by the hybrid material from ethanol–water mixture was carried out by putting a solid sample of 0.02 g in contact with 20 mL of a 6 g L^−1^ cesium nitrate in a 4:1 ethanol–ultrapure water mixture under reflux for 5 h. The volume ratio between the two solvents was chosen to be the same as in the case of ion exchange with ammonium so as to ensure sufficient solubility of hexadecylamine units displaced from the interlayer space to the bulk solution. The final solid was separated from the supernatant solution by centrifugation at 11,000 rpm for 10 min, washed with ethanol and water, and then dried at 343 K. The ICP-OES and elemental analysis were employed to determine the cesium, carbon, and nitrogen contents directly in the solid phase before and after sorption. The ICP-OES configuration used for the analysis of cesium after dissolution of the solid sample in concentrated nitric acid was similar to that described above, with the sole differences in wavelengths (455.531 nm for cesium) and in numbers of replicates (4 for cesium).

The sorption capacity and affinity of vanadium oxide materials toward cesium in aqueous solutions were studied by measuring the individual sorption isotherms under the unadjusted pH condition. A 0.020-g solid sample was first placed into a Nalgene™ reactor and 20 mL of cesium nitrate solution having a concentration varying from 0.05 mmol L^−1^ to 4 mmol L^−1^ were added. To attain the sorption equilibrium, the tubes were stirred on a rotary shaker overnight at 298 K. The solid phase was subsequently separated from the liquid supernatant by centrifugation at 11,000 rpm for 10 min. After a filtration step making use of filters with a porosity of 0.22 µm, the supernatant was analyzed by high-performance liquid chromatography (HPLC). The supernatant composition was determined with a Shimadzu device composed of a Shim-pack IC-C1 column and a CDD-6A conductivity detector at 313 K. The volume of injection was equal to 45 μL and the flow rate 1.5 mL min^−1^. The mobile phase employed was composed of nitric acid (5 mmol L^−1^). The experimental uncertainties of the sorption measurements were assessed by following the previously established methodology (c.f., Electronic Supplementary Material for Ref. [40]). The maximum percentage error was within 8% in the sorption plateau region. When plotting the experimental sorption isotherms, the error bars used to indicate the uncertainty in the sorption experiments refer to this maximum value.

In artificial seawater, the equilibrium concentration of cesium was determined by inductively coupled plasma mass spectrometry (ICP-MS). The collected samples were analyzed by mass spectrometry with a plasma source on quadrupole ICP-MS (Agilent 7700×) available at the Platform for Trace Elements Analysis in Environment & Isotopes (AETE-ISO) of the University of Montpellier. The ^133^Cs isotope was chosen for the determination of cesium. The apparatus was equipped with Micro concentric Glass Nebulizer operating at a flow rate of 200 µL min^−1^. The radiofrequency power was fixed at 1500 W. The collision cell was purged with ultrapure helium (purity > 99.999%). On the day of analysis, the samples were diluted in 2% nitric acid in order to adapt the concentration to the detection range of the device. The dilution factors considered here ranged from 10,000 to 30,000. Using large dilution factors allowed minimizing the instrumental drift related to the matrix (sea water), the latter being corrected using two standards (^115^In and ^209^Bi) in the samples. The elements were analyzed and their concentrations quantified by external calibration through multi-elementary synthetic solutions prepared daily from mono-component solutions (concentrations ranging from 1 to 20 ppb). The reproducibility and the sensibility of the analyses were controlled by repeated measurements making use of the reference natural materials (SLRS6), prepared by following the same preparation procedures. The acquisition parameters were as follows: integration time per point—300 ms, number of points—3, replicates—3. 

The amount, *n_ads_*, of cesium units adsorbed onto solid was calculated from the initial solute concentration in each tube, *C_i_*, its equilibrium concentration, *C_eq_*, mass of the adsorbent, *m_S_*, and the initial volume of solution, *V*_0_, according to the following equation:

*n_ads_* = *V*_0_·(*C_i_* − *C_eq_*)/*m_S_*, (1)

The enthalpy changes accompanying the sorption of cesium from single-component nitrate solutions at 298 K were measured by means of an Isothermal Titration Calorimeter TAM III (TA Instruments, Guyancourt, France). The operating procedures and data processing are detailed elsewhere [40]. The enthalpy values corresponding to successive injections were summed up to obtain the cumulative enthalpy of displacement. This quantity was plotted against the relative amount of cesium retained at the solid–liquid interface. The latter was calculated from the related sorption isotherm by dividing the quantity of cesium sorption by the maximum sorption capacity of the solid sample. The repeatability of the enthalpy measurements was within 10%.

### 2.5. Simulation and Prediction of the Structure of Vanadium Oxide Materials

The computational effort was made to determine the structure models for the materials studied as a function of the interlayer cation. The initial atomic coordinates of the framework were taken from a refined structure obtained by XRD analysis of a material having a close chemical composition and reported previously [41]. Among the three lattice constants of the unit cell, the lengths *a* and *b* were fixed on the basis of the XRD data taken from the literature, whereas the length *c* was inferred from the XRD diffractograms recorded in the present work. The angle parameter *β* was adjusted so as to reproduce correctly the first main peak in the XRD pattern. Plausible crystallographic structures under different conditions were then proposed with the imposed C2-symmetry, in line with the results reported in the literature [41]. Furthermore, some OH groups were removed from this model to reproduce the experimental chemical composition and, in particular, the ratio between vanadium(+4) and vanadium(+5). The theoretical XRD patterns were thus generated from the simulated structures by means of a Reflex software. The comparison between the experimental and simulated patterns allowed the chemical structure and composition of the synthesized solids to be confirmed thoroughly.

The textural parameters of the simulated structures were estimated using the strategy previously reported by Düren et al. [42]. The accessible surface area was calculated from the area defined by the motion of the center of a nitrogen molecule rolling along the surface. The diameter of the probe molecule was considered to be equal to 3.681 Å, whereas the diameter of each atom constituting the vanadium oxide structure was taken from the UFF (Universal Force Field) data [43]. The pore volume was calculated for each simulated structure by using a similar method of trial insertions within the entire volume of the unit cell. A 0 Å-size probe was used to allow the determination of the free volume of the unit cell unoccupied by the framework atoms [42].

The computation to predict the IR spectra of molecules and solids was performed with the aid of the DFT code CASTEP [44] based on a geometry optimization using the GGA-PBE exchange correlation function [45] combined with the core-valence interactions described by ultra-soft pseudopotentials. The wave functions were expanded on a plane wave basis set with a kinetic energy cutoff of 400 eV.

## 3. Results and Discussion

To achieve the primary objective of the present study, it was first necessary to prepare vanadium oxide nanotubes having a scroll-like morphology and possessing hexadecylamine units intercalated between the rolled sheets. The intercalation of amine into the vanadium oxide layers had been previously demonstrated to occur during the maturation step after the hydrolysis of vanadium oxide dispersed in the amine solution and before hydrothermal treatment [46]. The formation of well-developed vanadate nanotubes during a two-step synthesis usually required the hydrothermal reaction to be conducted at 180 °C for at least 7 days [31,37,47]. In the present work, the appropriate chemical reactions were speeded up owing to the microwave-assisted hydrothermal process applied, thus resulting in a drastic shortening of the time for hydrothermal treatment to only a few hours.

**Figure 1:**
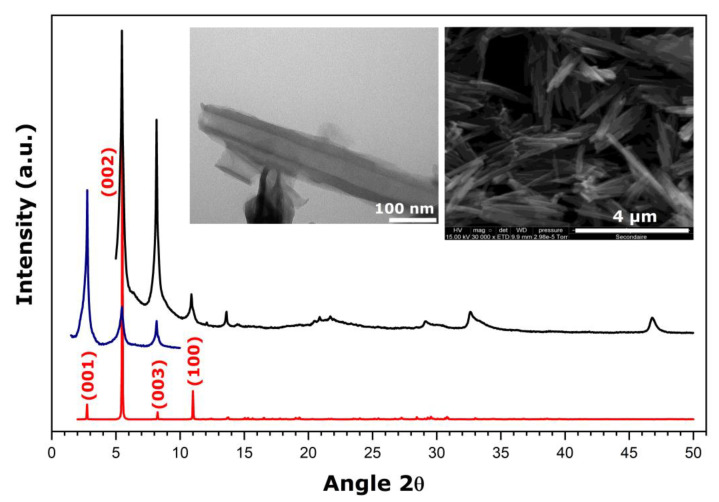
Theoretical XRD diffractogram simulated for a crystallographic structure without defects and a perfect stacking of the layers (**red line**) compared to room temperature wide-angle (**black line**) and small-angle (**blue line**) XRD patterns of the hybrid hexadecylamine-vanadium oxide material with a scroll-like morphology. The insets show TEM (**left panel**) and SEM (**right panel**) micrographs of the sample. This example corresponds to the sample prepared under synthesis conditions (right) detailed further in Table 2.

In view of establishing relevant criteria for deciding whether a given material has an appropriate layered structure and nanotube morphology on which to base future material selection, the XRD diffractogram was simulated for a crystallographic structure without defects and a perfect stacking of the layers. It should be noted that this structure has been built using a double unit cell with respect to the stacking parameter *c*. The resulting diffractogram is shown in Figure 1 and compared with typical XRD patterns recorded for one of the hybrid samples with a scroll-like morphology obtained within the framework of the present study.

The presence of diffraction peaks at small angles is explained by the large interlayer distance imposed by the presence of amine units in the structure. The interlayer distance, corresponding to the interlayer space opening together with the layer thickness, is equal to 32.07 Å, as inferred from the (001) peak in the XRD pattern in Figure 1. A good agreement between the experimental and simulated diffractograms provides evidence for a layered-type structuration in the form of a C2 monoclinic structure with the following cell parameters: *a* = 7.574 Å, *b* = 6.661 Å, *c* = 69.500 Å, and *β* = 111.29°. The SEM and TEM micrographs (the insets of Figure 1) reveal the nanotube morphology of solid particles which appear quite homogeneous in size. The multilayered wall structure that corresponds to a parallel arrangement of inorganic sheets shows up on the TEM image.

### 3.1. Optimization of the Synthesis Parameters to Achieve Vanadium Oxide Nanotubes

The following synthesis parameters were considered to achieve hybrid vanadium oxide nanotubes: (i) vanadium-to-amine molar ratio, (ii) aging time at 298 K, and (iii) duration of the microwave-assisted hydrothermal treatment at 463 K. Their values taken for comparison are reported in Table 2 below.

**Table 2:** Synthesis parameters varied to achieve hybrid vanadium oxide nanotubes.

Sample	Vanadium-to-Amine Molar Ratio	Aging Time at 298 K (Days)	Duration of the Hydrothermal Treatment at 463 K (Hours)
(a)	2	3	2
(b)	2.5	3	2
(c)	4	3	2
(d)	2.5	5	2
(e)	2.5	2	2
(f)	2.5	5	1

**Figure 2:**
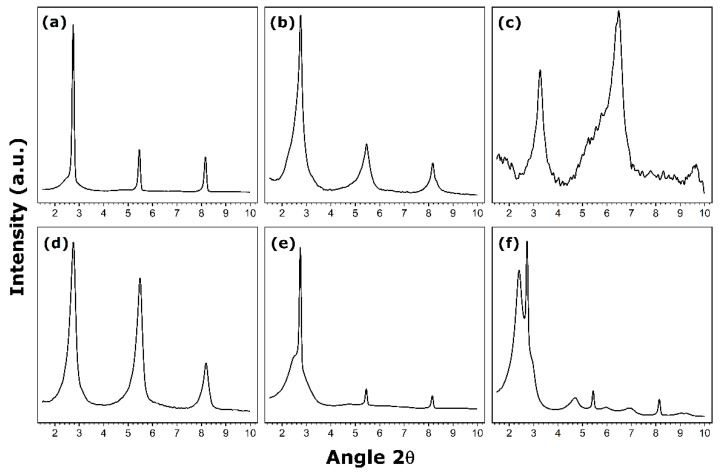
Small-angle XRD patterns of hybrid hexadecylamine-vanadium oxide materials prepared under the synthesis conditions detailed in Table 2 (each letter indicating a given panel corresponds to that in Table 2).

Figure 2 shows the small-angle X-ray diffraction patterns for hybrid materials prepared under the synthesis conditions specified in Table 2.

**Figure 3:**
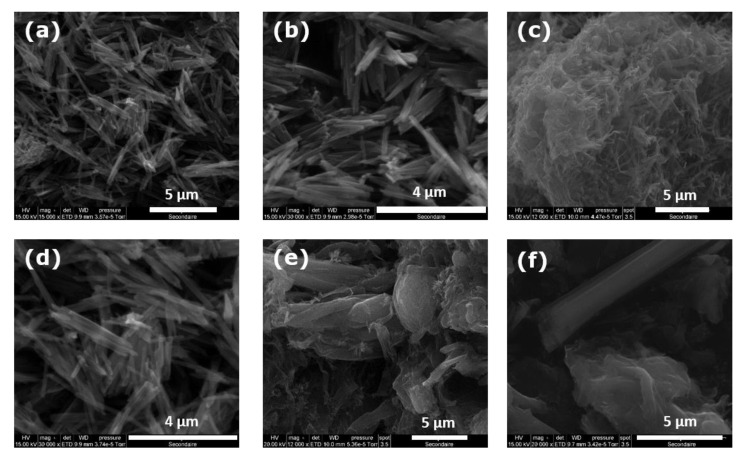
SEM micrographs of hybrid hexadecylamine-vanadium oxide materials prepared under the synthesis conditions detailed in Table 2 (each letter indicating a given panel corresponds to that in Table 2).

The related SEM micrographs are given in Figure 3 to support the conclusions drawn from the analysis of the XRD patterns.

For the vanadium-to-amine molar ratio being equal to 4, the nanotube structures are not well-defined, as the XRD reflections at small angles are greatly broaden (Figure 2c). These broad reflections may be regarded as splitting into several contributions located close to each other, thus leading to important peak overlapping. It may be argued that the material is a mixture of various co-existing phases including nanotubes and other lamellar species (Figure 3c).

Increasing the concentration of amine to obtain a vanadium-to-amine ratio of 2.5 results already in well-defined nanotube structures (Figure 2b and Figure 3b). Further change of this ratio to 2 leads to the formation of a unique phase containing fully structured nanotubes, characterized by the thin reflections in the XRD pattern (Figure 2a and Figure 3a). These trends confirm again that the presence of the structure directing hexadecylamine in appropriate quantities during the maturation stage is a pre-requisite for obtaining a multilayered wall structure. The latter corresponds to a parallel arrangement of vanadate sheets with the template units intercalated between them. Nevertheless, it should be also remembered that an increase in the amount of amine units inserted in the layered structure greatly enhances its hydrophobic character, thus precluding its use in water remediation.

The aging stage at room temperature lasting for 2, 3, or 5 days may also affect the structuration of vanadium oxide nanotubes mainly due to slow diffusion of the amine units inside the lamellar structure and their interactions with the layered framework dominated by electric and Van der Walls forces [33,36]. For a suspension aged during 2 days, the XRD pattern in Figure 2e again suggests the presence of a phase mixture, since the first reflection at small angles appears to split into two contributions. In contrast, after 3 days of aging, the presence of three clearly defined peaks in the XRD pattern in Figure 2b argues in favor of well-structured nanotubes. When the suspension is aged for 5 days, the nanoroll structure does not seem to have improved since the XRD pattern in Figure 2d is very similar to that recorded after 3 days of aging. As a conclusion, an optimal aging of 3 days is necessary to obtain well-defined vanadium oxide nanotubes.

As far as the microwave-assisted hydrothermal treatment is concerned, the XRD pattern of the material irradiated with microwaves only during 1 h is shown in Figure 2f. This duration is clearly insufficient to achieve the expected nanoroll structure and it should be prolonged to 2 h (Figure 2d).

### 3.2. Textural Properties of the Selected Hybrid Layered Structure and Effect of Ion Exchange on Its Structural Properties and Morphology

In view of testing the capacity and affinity of hybrid materials toward cesium removal, the first step was to render them less hydrophobic and thus hinder the unwanted aggregation of particles in polar media which would greatly decrease the surface of contact between the solid phase and the solution. On the other side, the goal was to preserve as much as possible the layered structure and the nanotube morphology. For this reason, the nanotube samples corresponding to synthesis conditions (a), (b), and (d) were first considered. Finally, it was decided that the hybrid material (b) represented the best compromise between the lowest hexadecylamine content (i.e., vanadium-to-amine molar ratio of 2.5), still enough to obtain the desired nanostructuration, and the shortest synthesis time (aging step during 3 days). This material further used in sorption studies is referred to as VO_x_-C16.

In order to complete the knowledge of the selected sample, the contents of carbon and nitrogen constituting the organic phase were determined by elemental analysis with respect to the atomic percentage of vanadium, as inferred from ICP-OES measurements. Moreover, XPS studies made on this sample provided the oxidation number of vanadium corresponding to 34% of vanadium(+4) in the form of VO_2_ and 66% of vanadium(+5) as V_2_O_5_. The combination of these results allowed the chemical formula of the sample to be proposed as (V_2_O_5_)(VO_2_)_1.03_(C_16_H_36_N)_1.46_(H_2_O)_x_, with x denoting the hydration degree of the vanadium oxide layers.

**Figure 4:**
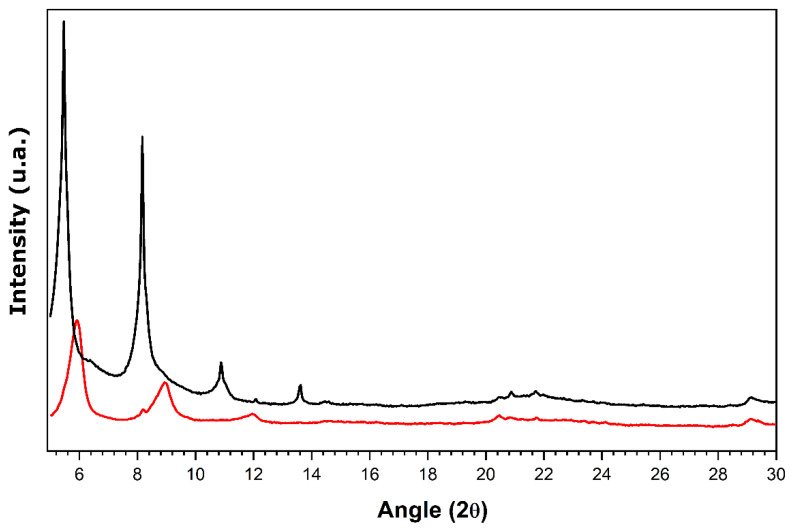
Room temperature XRD patterns of hybrid hexadecylamine-vanadium oxide material, VO_x_-C16, recorded under ambient air (**black line**) and vacuum (**red line**) conditions.

The structural flexibility of VO_x_-C16 was first tested under low-pressure conditions resulting in dehydration of the sample. For this purpose, an additional XRD study was performed by subjecting the sample to vacuum in the vacuum chamber of the diffractometer. Room temperature XRD patterns recorded with this sample under ambient air or vacuum conditions are shown in Figure 4.

A significant shrinkage of the interlayer space and, to some extent, amorphization of the structure due to the departure of water molecules in vacuum may be clearly inferred from the increase in the (001) peak position. The observed effect argues in favor of some structural flexibility of the layered framework containing the amine units incorporated within the interlayer space. This shows the potential of the interlayer space to adjust itself to the dimension of the inserted species.

On the other side, the impossibility of sample dehydration by vacuum thermal treatment without compromising the solid structure precludes the use of N_2_ adsorption to determine the textural parameters of VO_x_-C16, as is routinely reported in the literature [48]. In consequence, these parameters have been only estimated in the present study based on Monte Carlo molecular simulations. The results are given in Table 3.

It has been proposed that the amine molecules are protonated during the synthesis and the cationic C_16_H_36_N^+^ species remain intercalated within the oxide layers [46,49]. In such a case, the hybrid hexadecylamine-vanadium oxide material should possess some cation exchange capacity in a solvent which is capable of dissolving amine units.

In the case of clay minerals, the cation exchange capacity has been usually studied with ammonium saturated clays [50,51]. In the present work, similar principle was followed and ammonium cation, NH_4_^+^, was chosen to displace amine units, C_16_H_36_N^+^, from the interlayer space. To increase the efficiency of such an exchange procedure, it was carried out by making use of a 4:1 ethanol–water mixture as solvent in which the released amine units were soluble. The choice of NH_4_^+^ as the counter-ion in the ultimate material was also justified by the radius of the hydrated cation, r_hyd_, and the enthalpy of hydration in the bulk, ΔH_hyd_, water being close to those of the targeted Cs^+^ ion (i.e., r_hyd_ = 213 pm, ΔH_hyd_ = −325 kJ mol^−1^ for NH_4_^+^ and r_hyd_ = 219 pm, ΔH_hyd_ = −280 kJ mol^−1^ for Cs^+^ [52]). The exchange process was carried out progressively by repeating the same exchange procedure. According to the results of elemental analysis, the exchange ratio after the first exchange step was about 70% and three subsequent exchange procedures were necessary to almost completely eliminate the hexadecylamine template from the interlayer space.

**Figure 5:**
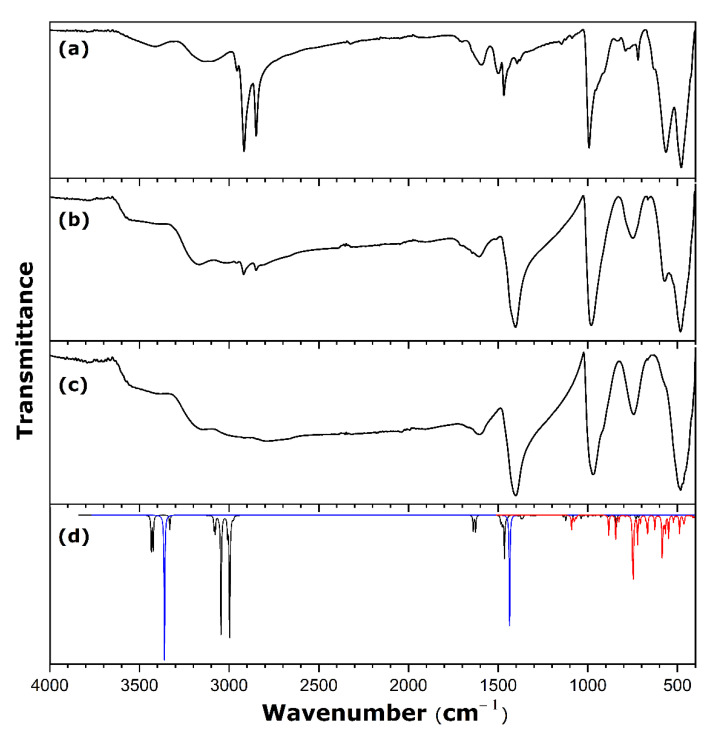
Experimental infra-red spectra of the pristine and exchanged samples: (**a**) hybrid hexadecylamine-vanadium oxide material, VO_x_-C16, (**b**) pristine sample after one exchange with NH_4_^+^, (**c**) pristine sample after two-stage exchange with NH_4_^+^, (**d**) simulated IR spectra of protonated amine cation (black line), NH_4_^+^ ion (blue line), and model vanadium oxide structure containing NH_4_^+^ (red line).

Figure 5 shows infrared spectra of the pristine material and two exchanged samples. In order to determine unambiguously the position of various vibration bands, the experimental spectra are confronted with the simulated IR spectra of protonated amine cation, NH_4_^+^ ion, and layered vanadium oxide containing NH_4_^+^ units in the interlayer space (Figure 5d).

The IR spectrum of the pristine sample obtained in the present study (Figure 5a) is similar to those reported in the literature for analogous materials loaded with amine [32,53,54]. According to Figure 5d where the IR bands specific to the amine cation appear around 1500 and 3400 cm^−1^, the vibrations at 2959, 2921, 2851, 1493 1469, and 1399 cm^−1^ can be assigned to the C-H vibrations of hexadecylamine species. The vibrations at 3415, 3122, and 1604 cm^−1^ are attributed to the N-H vibrations of the amine. The frequency at 3415 cm^−1^ is to be attributed to the O-H vibrations, thus illustrating the intercalation of water molecules in interaction with the solid surface. The frequencies at 995, 794, 721, 568, and 479 cm^−1^ correspond to the vanadium-oxygen bond, as corroborated by the simulated IR spectra (c.f., the red bands in Figure 5d). The signature of vanadium oxide structure is mainly present in the range of 400–1000 cm^−1^ (c.f., Figure 5d) [53,54]. It is worth noting that the bands corresponding to hexadecylamine disappear progressively when passing from Figure 5a to Figure 5c. Within the range of detection limit of the IR infrared spectrophotometer, the sample obtained after two exchange steps with ammonium cations already seems free from the hexadecylamine template.

The chemical composition of the vanadium oxide structure obtained after the last exchange step was again investigated by coupling ICP-OES, XPS, and elemental analysis. The vanadium content was about 50 wt.% splitting into 34 wt.% of vanadium(+4) and 65 wt.% of vanadium(+5), in a good agreement with the pristine composition. The carbon content was about 1.8 wt.%, thus indicating that a small amount of amine remained in the sample. In consequence, the chemical formula of this sample was as follows: (V_2_O_5_)(VO_2_)_0.53_(C_16_H_36_N)_0.01_(NH_4_^+^)_0.34_.

**Figure 6:**
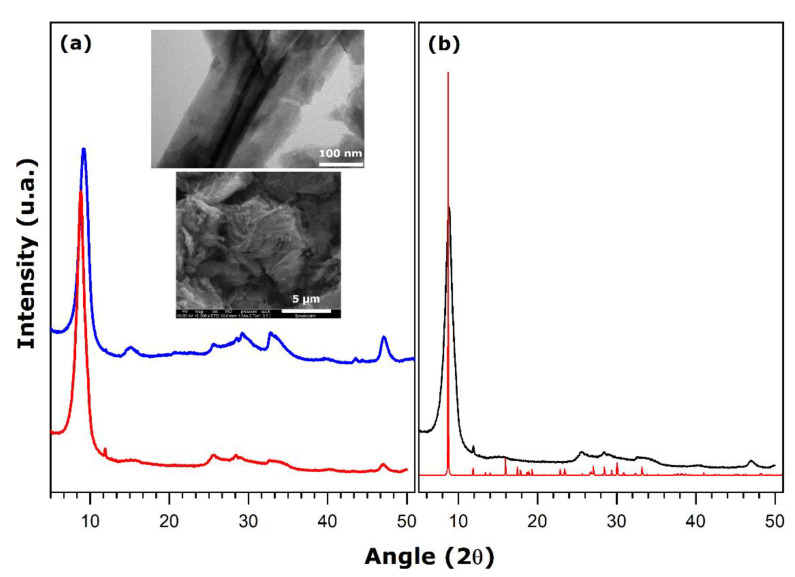
Wide-angle XRD patterns recorded on hybrid hexadecylamine-vanadium oxide samples exchanged with ammonium cation: (**a**) after the first (**blue line**) and the second (**red line**) exchange step, (**b**) simulated diffractogram (**red line**) generated for the theoretical vanadium oxide structure modified to take into account the real stoichiometry and containing exclusively NH_4_^+^ cations in the interlayer space in comparison with the experimental one (**black line**) after the third exchange step. The inset shows TEM (upper image) and SEM (lower image) micrographs of the sample after one exchange.

The cation exchange capacity of VO_x_-C16 was assessed by paying particular attention to potential changes in the layered structure and scroll-like morphology accompanying such a process. Some evidence of significant structural evolution is given in Figure 6 where the XRD patterns of the three exchanged materials are confronted to the diffractogram simulated for a monoclinic C2-structure with the following cell parameters: *a* = 7.574 Å, *b* = 6.661 Å, *c* = 20.650 Å, and *β* = 100.290° (the double cell according to *c*-parameter).

The SEM and TEM micrographs in Figure 6 unambiguously point out that the nanoscale scroll-like structure has been partially altered after one exchange. Given additionally the XRD patterns reported in Figure 6, it can be concluded that the vanadium oxide layers undergo an unrolling process and only the lamellar structures survive after all exchange steps. As can be inferred from the analysis of the (001) peak in the XRD patterns of the as-obtained (Figure 1) and once-exchanged samples (Figure 6a), the interlayer distance changes from 32.07 Å to 9.65 Å. After two exchanges, this distance is about 9.97 Å. Clearly, the structure relaxation occurs when the nature of the interlayer cation present in the interlayer space is modified, like in the case of swelling clays [55].

Finally, the same molecular simulation procedure as in the case of VO_x_-C16 sample was used to evaluate the specific surface area and pore volume of the hypothetical layered structure obtained with NH_4_^+^ cations. The results have been added to Table 3. The comparison of the values reported in Table 3 for both structures confirms the significant decrease in the textural parameters, and particularly in the pore volume upon replacement of C_16_H_36_N^+^ by NH_4_^+^.

### 3.3. Testing Cesium Retention Performance of the Pristine VOx-C16 and Once-Exchanged Sample in Polar Media

In the first step, a one-point test of cesium retention by VO_x_-C16 was carried out by putting a solid sample in contact with a concentrated solution of cesium nitrate in a 4:1 ethanol–water mixture. The ICP-OES and elemental analysis were then employed to determine the cesium, carbon, and nitrogen contents directly in the solid phase before and after sorption (Table 4). Note that the element contents are expressed as mass/weight percent.

According to the results reported in Table 4, the amount of cesium entrapped within the layered structure is about 2.9 mmol g^−1^. This capacity of cesium uptake places the sample among the best performing nanomaterials available [28]. The significant decrease in the carbon content corroborates the release of hexadecylamine units to the ethanol–water mixture. Based on the mass balance between the two states, the cation exchange pathway involving C_16_H_36_N^+^ and Cs^+^ ions is responsible for 65% of the total cesium uptake (~1.9 mmol g^−1^). The decrease in the nitrogen content does not follow that of carbon in exactly stoichiometric proportions and it appears somewhat enhanced. This suggests that some other nitrogen-containing species are loaded on the vanadium oxide material. Since the final structure should remain electrically neutral, it is likely that one part of cesium is retained together with its nitrate counter-ion (NO_3_^−^). Indeed, the mass balance of the samples before and after sorption indicates that about 1 mmol g^−1^ of CsNO_3_ has been inserted in the final material.

This uptake of the neutral species may be rationalized on the basis of a decrease in the solubility of CsNO_3_ in ethanol–water mixtures, thus increasing the probability of ion pairing [56]. Furthermore, such an association between Cs^+^ and NO_3_^-^ should be certainly enhanced when hexadecylamine units are progressively released to the surrounding ethanol–water mixture.

**Figure 7:**
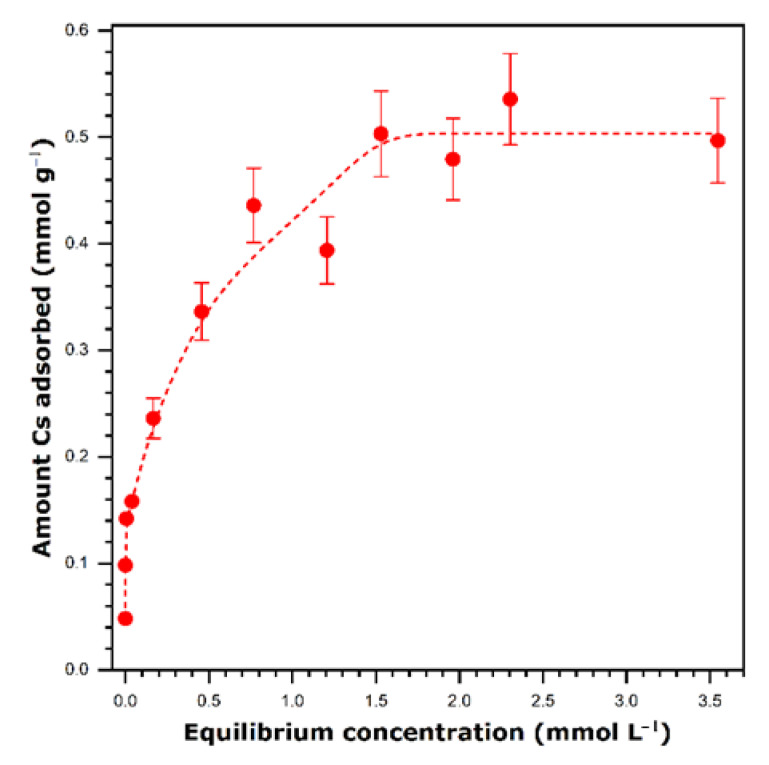
Sorption isotherm for cesium cations onto the hybrid hexadecylamine-vanadium oxide, VO_x_-C16, sample from single-component solutions in ultrapure water at 298 K. The error bars represent, for selected points, the maximum percentage error in the sorption experiments. The dashed line is drawn to guide the eye.

In the context of decontamination of nuclear waste, it is more relevant to consider the removal of cesium from aqueous streams. Therefore, an isotherm of cesium sorption onto VO_x_-C16 from cesium nitrate solutions in ultrapure water was measured by solution depletion method. The results are presented in Figure 7. According to the classical speciation diagram [28], cesium primarily forms free Cs^+^ cations in aqueous media.

Compared to the case of sorption from alcohol–water mixture, cesium uptake from ultrapure water decreases to a great extent. The maximum retention capacity of VO_x_-C16 toward this metal can be estimated at 0.50 ± 0.05 mmol g^−1^.

In order to shed more light on the sorption mechanism, Table 5 reports the carbon and nitrogen contents in the sample before and after sorption, as determined by elemental analysis for one point on the sorption plateau.

Contrary to the results reported in Table 4, nothing seems to change at a first glimpse since the carbon and nitrogen contents may be considered, within the experimental error, as being the same before and after sorption. Nevertheless, 0.5 mmol of cesium per unit mass of the final sample has been added, which should have resulted in a decrease in the content of other elements. Surprisingly, the percentage of carbon in the sample rather appears to be enhanced in comparison with the nitrogen content after sorption of cesium. Given a more significant uncertainty in the measurement of the carbon content, further simulations will be done by normalizing the composition with respect to the percentage of nitrogen. If the sorption mechanism had been limited only to a stoichiometric exchange, between Cs^+^ and C_16_H_36_N^+^, of 0.5 mmol per unit mass of the final sample, the mass of the solid material would have decreased from 100 g to 94.81 g after reaching the sorption equilibrium. Simultaneously, the mass of the carbon would have changed from 34.3 g (i.e., the mass corrected in relation with the nitrogen percentage in Table 5) to 25.2 g, thus giving a final carbon content of 26.6 wt.%. The percentage of nitrogen would have been 1.9 wt.%. These results at variance with the values reported in Table 5 argue against the direct exchange between cesium cations and hexadecylammonium ones. This is mainly due to very low solubility of hexadecylammonium salts in water at ambient temperatures [57], which hinders the displacement of the protonated amine units from the interlayer space.

Rather, it can be suggested that a carbon-containing compound is retained by VO_x_-C16. The most plausible explanation is that the sorption of cesium within the interlayer space occurs as Cs_2_CO_3_ taking the form of a neutral compound or an ion-pair complex. Indeed, the carbonate species are present in aqueous solutions since the pH of ultrapure water used is about 5.8 due to the dissolution of atmospheric CO_2_.

Furthermore, cesium carbonate is characterized by an important solubility in organic solvents much greater than that of potassium or sodium analogues [58]. Following the above mentioned calculation procedure, the mass of the solid sample is predicted to increase from 100 g to 108.9 g and the corresponding nitrogen and carbon contents are 2.3 wt.% and 32 wt.%, respectively. They correspond better to the experimental values reported in Table 5, thus validating the hypothesis of Cs_2_CO_3_ uptake by VO_x_-C16.

In order to much increase the sorption of cesium from aqueous solutions, one of the exchanged samples should rather be used in further study. The decreased hydrophobic character of the solid sample and the capacity of preserving at least the layered structure were the key factors in selecting the right material. Therefore, the choice of sample was oriented toward VO_x_-C16 after one-step exchange with NH_4_^+^. It is further referred to as VO_x_-E1.

**Figure 8:**
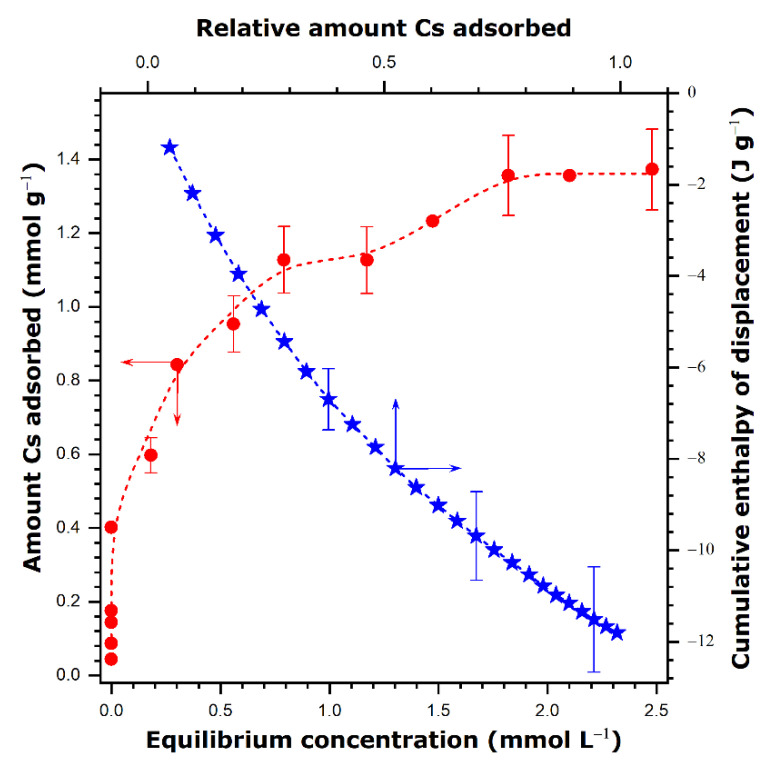
Sorption isotherm (**red circles**) for cesium cations onto VO_x_-E1 sample from single-component solutions in ultrapure water at 298 K and the corresponding cumulative enthalpy of displacement curve (**blue stars**). The error bars represent, for selected points, the maximum percentage error in the sorption experiments and the repeatability of the enthalpy measurements. The dashed lines are drawn to guide the eye.

The cesium sorption isotherm from single-solute solution in ultrapure water and the cumulative enthalpy of displacement curve are presented in Figure 8.

The sorption curve represents a high-affinity curve with a quasi-vertical initial portion at very low concentrations and a plateau sorption at high concentrations. The plateau value (1.4 ± 0.13 mmol g^−1^) gives the maximum sorption capacity of this sample toward cesium in aqueous solutions. According to Table 6, this retention performance is situated between those of manganese oxides and hexacyanoferrates. It is close to the cesium sorption capacity exhibited by a magnetic zeolite A composite or titanate nanotubes.

It is also possible to evaluate the affinity coefficient, K_D_, from the slope of the initial portion (the so-called Henry’s law constant) by using the initial linear part of the isotherm. For the sake of simplicity, the values of log(K_D_) are further reported in the text. In the case of the very first experimental points on the sorption curve, the HPLC detector yields a value of zero for the concentration of cesium in the equilibrium supernatant solution. Since this is due to the limited precision of the HPLC technique in this very low concentration range (i.e., the concentration values are below the detection limit), these very first points have not been taken into account when calculating the K_D_ value. Moreover, a more classical procedure based on a curve fitting to theoretically describe the experimental sorption data in the whole sorption range was avoided for two reasons. First, the simple isotherm equations frequently used in the literature to fit the high-affinity curves (e.g., Langmuir equation) are not consistent with the competitive mechanism of sorption at the solid–liquid interface. Second, the main intention here is to shed light on the affinity of the solid surface for cesium cations, which manifests itself in the very beginning of the individual sorption isotherm. Finally, the affinity constant K_D_ is equal to 5 thus indicating the strong affinity of VO_x_-E1 toward cesium.

The results of elemental analysis of the sample before and after sorption are given in Table 7 for one point lying on the sorption plateau.

If one assumes a cation exchange between Cs^+^ and NH_4_^+^ of 1.4 mmol per unit mass of the final sample, the theoretical carbon and nitrogen contents will be equal to 0.94 wt.% and 1.6 wt.%, respectively. Even though there is some discrepancy between the theoretical and experimental values, these results provide a strong indication that only the ammonium cation is exchanged with Cs^+^, while the protonated hexadecylamine units remain within the structure due to their low solubility in water. It is worth noting that the calculation performed on the basis of the nitrogen contents in Table 7 would result in a maximum cesium uptake of 1.6 mmol g^−1^.

The strong affinity of VO_x_-E1 toward cesium is evidenced simultaneously by very exothermic displacement effect in Figure 8. The enthalpy of displacement represents the total enthalpy balance upon sorption and it includes the effects due to ion exchange between the interface and the bulk solution, as well as the dehydration of the adsorbing cations and the rehydration of the one released to the solution [64,65]. Given the fact that the hydration enthalpies of NH_4_^+^ and Cs^+^ in the bulk water are of comparable magnitude [52], negative values of the enthalpy of displacement upon cesium sorption within the whole sorption range point out that the sorption affinity toward cesium cation is much stronger than that of ammonium one.

It is also worth noting that the maximum sorption capacity of VO_x_-E1 from single component aqueous solutions is far from that measured for VO_x_-C16 in the ethanol–water mixture. It is rather comparable, within the experimental error, to the contribution ascribed previously to the ion-exchange mechanism (i.e., ~1.9 mmol g^−1^). It is thus clear that the retention of cesium as CsNO_3_ is to be ruled out here and only the cation exchange is responsible for the sorption of free metal cations. Nevertheless, this does not necessarily mean that the capability of VO_x_-C16 to uptake CsNO_3_ is due to its scroll-like morphology, being more pronounced than in the case of VO_x_-E1. Considering the available results, the great difference in the salt solubility in both types of liquid media is the most plausible explanation for the observed effect. No arguments for the synergic effect of the nanotube morphology in cesium sorption may be advanced on this basis.

**Figure 9:**
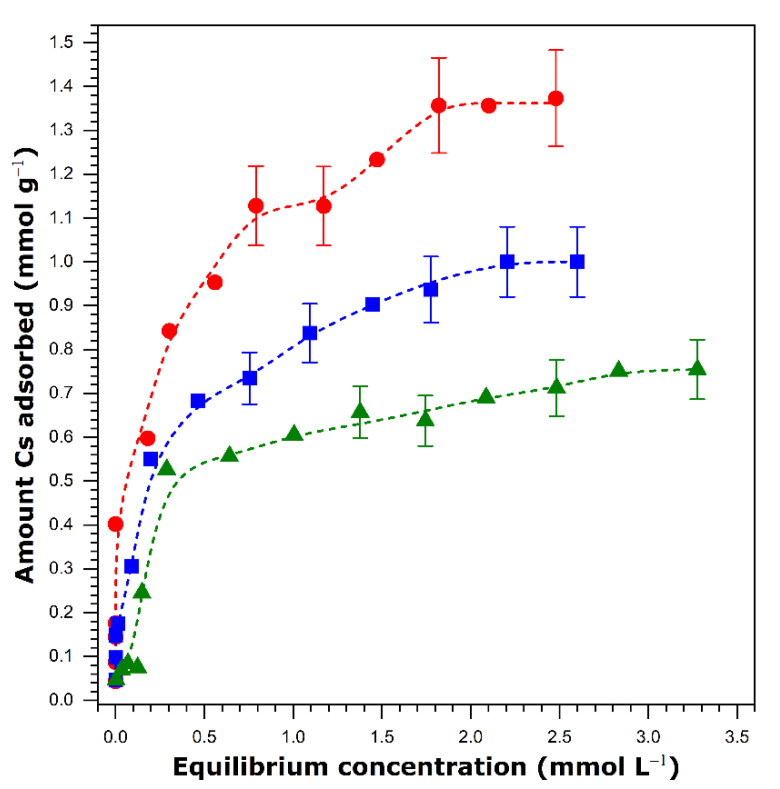
Sorption isotherms for cesium ions onto VO_x_-E1 sample from single solute solutions (red circles) and multicomponent aqueous solution corresponding to the Ca-rich mineral water (blue squares) or simulated sea water (green triangles). The error bars represent, for selected points, the maximum percentage error in the sorption experiments. The dashed lines are drawn to guide the eye.

Cesium sorption tests were performed also under conditions of competition among various species present in mineral and sea waters. The sorption isotherms determined making use of the same material VO_x_-E1 are shown in Figure 9.

Taking into account the sorption isotherm obtained with the use of VO_x_-E1 in single solute solutions as the reference (Figure 8), a 30% decrease in the maximum amount adsorbed from Ca-rich mineral water is observed in Figure 9 (1.0 ± 0.1 mmol g^−1^ instead of 1.4 ± 0.13 mmol g^−1^). Simultaneously, the value of log(K_D_) decreases from 5 to 4. These changes illustrate the effect of such competing cations as calcium, potassium, sodium, and magnesium on the sorption capacity and affinity of the vanadium oxide toward cesium. Even though the effect is pronounced in the adsorption plateau region, the sample still remains quite efficient in the cesium removal from aqueous solutions of cesium concentration up to about 0.3 mmol L^−1^. This is particularly important given the high concentrations of calcium (i.e., 1.57 mmol L^−1^) and magnesium (i.e., 0.42 mmol L^−1^) in mineral water. In this respect, it seems likely that the VOx-E1 material containing a mixture of C_16_H_36_N^+^ and NH_4_^+^ cations in the interlayer space is selective toward cesium against bivalent mineral cations.

In addition, the quantity of Cs^+^ sorption onto VOx-E1 was measured from multicomponent solutions simulating the composition of sea water. The sorption isotherm is also reported in Figure 9. The comparison of the sorption performance for the two types of multicomponent aqueous solution (i.e., Ca-rich mineral water and synthetic sea water) shows a further decrease in the saturation capacity from 1.0 ± 0.1 to 0.8 ± 0.1 mmol g^−1^. Moreover, the log(K_D_) value inferred from the steep portion of the sorption isotherm decreases from 4.0 to 3.5. When analyzing these changes, it is necessary to keep in mind the increased ionic strength of the aqueous phase mostly due to the very high concentration of sodium (i.e., 470 mmol L^−1^). Although the size of the hydrated Na^+^ cation (i.e., r_hyd_ = 218 pm) is comparable to those of Cs^+^ and NH_4_^+^, somewhat more energy is necessary to dehydrate it since ΔH_hyd_ = −415 kJ mol^−1^ [52]. On the other side, the bare Na^+^ ion is much smaller than Cs^+^ and NH_4_^+^ ones and, as such, it seems much less entrapped within the interlayer space of VO_x_-E1. As a consequence, the cation exchange between NH_4_^+^ and Cs^+^ should dominate over that between NH_4_^+^ and Na^+^ despite the overwhelming concentration of the latter in the aqueous phase. This conclusion is supported by the shape of the adsorption isotherm, at least in the range of cesium concentration up to about 0.3 mmol L^−1^. Finally, VO_x_-E1 appears selective toward cesium also against monovalent mineral cations.

## 4. Conclusions

Layered vanadium oxide materials containing hexadecylamonium cations or a mixture of hexadecylammonium and ammonium cations in the interlayer space have proven efficient for cesium removal from some polar liquid media. A microwave-assisted synthesis pathway was successfully employed to speed up the preparation of well-structured vanadium oxide samples with a nanotube morphology by making use of hexadecylammine as a structure directing agent. A good compromise between the scroll-like morphology, the duration of the synthesis route, and the hydrophobic character of the as-synthesized hybrid material was obtained for the experimental conditions corresponding to a vanadium-to-amine molar ratio of 2.5, an aging time of 3 days at room temperature, and two-hour hydrothermal treatment at 463 K. The material was demonstrated to be capable of retaining up to 2.9 mmol of cesium per unit mass of the sample from a 4:1 mixture of ethanol and water. About two-thirds of this sorption capacity was achieved through ion exchange between the hexadecylammonium cations and free metal ones. The remainder was attributed to the sorption of CsNO_3_ induced by the decreasing solubility of the salt in the supernatant solution. As studied in view of more relevant applications, the sorption of cesium from single component aqueous solutions was greatly reduced to only 0.5 mmol g^−1^, thus arguing in favor of metal uptake within the interlayer space in the form of Cs_2_CO_3_. The subsequent removal of about 70% of the hexadecylammine template and its replacement by ammonium cations resulted in a material better dispersible in aqueous solutions but representing a stacking of lamellar sheets with a much worse scroll-like morphology. Its maximum sorption capacity toward cesium from single component solutions in ultrapure water was about 1.4 mmol g^−1^ corresponding solely to ion exchange between ammonium cations and free cesium ones. The effective retention of cation by this exchanged sample was found to be still significant in multicomponent aqueous solutions in which the cesium concentration was smaller than 0.3 mmol L^−1^, even those having a very complex composition to simulate river or sea water.

It appears from the present study that the nanotube morphology does not necessarily need to accompany the layered structure to achieve vanadium oxide-based materials with enhanced retention capacity toward cesium from aqueous and other polar media. Within the framework of cation exchange mechanism, this retention capacity is chiefly governed by the solubility of the cationic species displaced by the oncoming cesium ions from the interlayer space to the equilibrium bulk solution.

In parallel, the results of cesium sorption by layered vanadium oxide provide another perspective on potential applications in catalysis or energy conversion. Some significant but non-exhaustive examples can be provided to illustrate the use of cesium-doped vanadium oxide as the active phase (or a part of it) of an efficient catalyst for catalytic decomposition or oxidation [66,67,68], as well as thin films with high electrical conductivity in perovskite solar cells [69].

## Figures and Tables

**Table 1:** Composition of multicomponent aqueous solutions used to prepare cesium solutions for the purpose of retention tests [38].

Ion	Mineral Water Rich in Ca^2+^ (mmol L^−1^)	Synthetic Sea Water (mmol L^−1^)
	pH = 7.6	pH = 10.3
Ca^2+^	1.57	10.3
Mg^2+^	0.42	53
Na^+^	0.06	470
K^+^	0.01	10.2
HCO_3_^−^	2.84	1.66
SO_4_^2−^	0.53	28
NO_3_^−^	0.03	-
Cl^−^	-	550

**Table 3:** Specific surface area (SSA) and pore volume (PV) accessible to N_2_ molecules calculated by molecular simulations for the layered vanadium oxide structures in which the distance of the empty interlayer space has been imposed equal to the experimental value as a function of the intercalated species.

Intercalated Species	SSA (m^2^ g^−1^)	PV (cm^3^ g^−1^)
C_16_H_36_N^+^	1470	1.68
NH_4_^+^	642	0.35

**Table 4:** Cesium, carbon, and nitrogen contents (in percent by mass) of the hybrid hexadecylamine-vanadium oxide, VO_x_-C16, material before and after sorption from a 4:1 ethanol–water mixture.

Element	Content in the Sample (wt.%)	
	Before Sorption	After Sorption
Cesium	0	38.00 ± 0.45
Carbon	35.76 ± 0.02	0.02 ± 0.02
Nitrogen	2.59 ± 0.02	0.23 ± 0.04

**Table 5:** Carbon and nitrogen contents (in percent by mass) of the hybrid hexadecylamine-vanadium oxide, VO_x_-C16, material loaded with the maximum quantity of cesium before and after sorption from aqueous CsNO_3_ solutions.

Element	Content in the Sample (wt.%)	
	Before Sorption	After Sorption
Carbon	33.2 ± 1	34 ± 1
Nitrogen	2.5 ± 0.2	2.3 ± 0.2

**Table 6:** Sorption performance of some representative materials toward cesium in aqueous solutions.

Material	Sorption Capacity (mmol g^−1^)	Reference
Manganese oxide	2.0	[59]
Magnetic zeolite A composite	1.56	[60]
Titanate nanotubes	1.5	[61]
Phosphate-modified montmorillonite	0.71	[62]
K_2_[CoFe(CN)_6_]	0.5	[63]

**Table 7:** Carbon, and nitrogen contents (in percent by mass) of the hexadecylamine-vanadium oxide material after one exchange with NH_4_^+^, as corresponding to the plateau sorption value in Figure 8.

Element	Content in the Sample (wt.%)	
	before Sorption	after Sorption
Carbon	1.06 ± 0.07	1.11 ± 0.12
Nitrogen	4.23 ± 0.01	1.27 ± 0.01

## Data Availability

Data are contained within the article.
